# Metformin suppresses the growth of colorectal cancer by targeting INHBA to inhibit TGF-β/PI3K/AKT signaling transduction

**DOI:** 10.1038/s41419-022-04649-4

**Published:** 2022-03-02

**Authors:** Qing Xiao, Jiani Xiao, Jiaqi Liu, Jiaxin Liu, Guang Shu, Gang Yin

**Affiliations:** 1 Department of Pathology, Xiangya Hospital, School of Basic Medical Sciences, Central South University, Changsha, 410013 China; 2 Department of Histology and Embryology, School of Basic Medical Sciences, Central South University, Changsha, 410013 China; 3 China-Africa Research Center of Infectious Diseases, School of Basic Medical Sciences, Central South University, Changsha, 410013 China

**Keywords:** Colorectal cancer, Cell growth, Target identification

## Abstract

Multiple evidence shows that metformin serves as a potential agent for Colorectal Cancer (CRC) treatment, while its molecular mechanisms still require detailed investigation. Here, we revealed that metformin specifically suppressed the proliferation of CRC cells by causing G1/S arrest, and INHBA is a potential target for metformin to play an anti-proliferation effect in CRC. We verified the oncogene role of INHBA by knocking down and overexpressing INHBA in CRC cells. Silencing INHBA abrogated the cell growth, while overexpression INHBA promotes the proliferation of CRC cells. As an oncogene, INHBA was aberrant overexpression in CRC tissues and closely related to the poor prognosis of CRC patients. In mechanism, INHBA is an important ligand of TGF-β signaling and metformin blocked the activation of TGF-β signaling by targeting INHBA, and then down-regulated the activity of PI3K/Akt pathway, leading to the reduction of cyclinD1 and cell cycle arrest. Together, these findings indicate that metformin down-regulates the expression of INHBA, then attenuating TGF-β/PI3K/Akt signaling transduction, thus inhibiting the proliferation of CRC. Our study elucidated a novel molecular mechanism for the anti-proliferation effect of metformin, providing a theoretical basis for the application of metformin in CRC therapy.

## Introduction

Colorectal cancer (CRC) is the third most common malignant neoplasm after breast cancer and lung cancer, and the second leading cause of cancer death [1]. In recent years, the incidence and mortality of CRC are still increasing, and the age of onset is getting younger [2]. At present, the treatment of CRC mainly includes surgery, radiotherapy, chemotherapy, targeted therapy, and immunotherapy [3]. Among them, surgical resection is the radical treatment of CRC, but it is limited to early treatment. Fluorouracil, oxaliplatin, and irinotecan, combined with bevacizumab are considered first-line treatments for CRC [4]. However, the combination therapy also has many adverse reactions, such as neurotoxicity, hepatotoxicity, and drug resistance [5–7]. Therefore, seeking therapeutic drugs is still an important task to ameliorate the prognosis of patients with CRC.

Metformin is a biguanide hypoglycemic drug extracted from Galega officinalis. As a first-line clinical drug in the treatment of type 2 diabetes, metformin plays a hypoglycemic effect mainly by suppressing liver gluconeogenesis and improving insulin sensitivity [8]. Epidemiological evidence showed that besides reducing plasma glucose, metformin has also achieved good results in the prevention and treatment of CRC [9–12]. The current research is predominantly focused on the decrease of protein synthesis by activating Adenosine 5′-monophosphate (AMP)-activated protein kinase (AMPK) and blocking mammalian target of rapamycin (mTOR) [13–15]. However, many studies believe that the role of metformin in CRC therapy is controversial, and further studies are needed to confirm its antitumor effect [16]. Therefore, it is necessary to supplement the experimental evidence of the inhibitory effect of metformin on CRC progression and its molecular mechanism.

As a secretory protein, Inhibin βA (INHBA) is a member of the TGF-β superfamily. In most cases, two INHBA subunits form activin A, which activates the downstream Smad2/Smad3 signal pathway by binding to serine/threonine kinase receptors, thus regulating cell growth and differentiation [17]. In recent years, more and more evidence showed that the high expression of INHBA was related to the occurrence and development of various malignant tumors, such as ovarian cancer [18, 19], gastric cancer [20], pancreatic cancer [21], and CRC [22].

In this study, we first found the regulatory relationship between metformin and INHBA in CRC. It was observed that high levels of INHBA in tumor tissues were associated with poor prognosis of CRC patients, and metformin could significantly inhibit the mRNA and protein expression of INHBA and down-regulate TGF-β/PI3K/Akt signal transductions to achieve an inhibitory effect on the proliferation of CRC. Our study confirmed that INHBA is a molecular target for metformin to suppress the proliferation of CRC, which provides new evidence for the application of metformin in CRC intervention.

## Materials and methods

### Clinical samples

We totally collected 138 pairs of paraffin specimens from patients diagnosed with CRC from 2011 to 2013. A total of 100 fresh CRC tissues and 58 adjacent noncancerous tissues were also acquired for quantitative real‐time polymerase chain reaction (qRT-PCR) and western blot analysis. All the above clinical samples were gained from Xiangya Hospital, Central South University (Changsha, China). This study has obtained informed consent from patients and was approved by the Protection of Human Subjects Committee of Xiangya Hospital.

### Animal experiments

We chose 5-week-old male BALB/c (nu/nu) nude mice for in vivo experiments according to the approved protocols. The mice were randomly divided into two groups and subcutaneously injected with 1 × 10^6^ HCT116 cells. After 5 days, the control and metformin groups were injected intraperitoneally with phosphate-buffered saline (PBS) and metformin (150 mg/kg body weight per day) for 3 weeks, respectively. Tumor volume (length × width^2^ × 0.5) was measured every 2 days. The protocols for animal care and euthanasia were approved by the Institutional Animal Care and Use Committee of Central South University (Changsha, China).

### Cell lines and cell culture

The human CRC cells LoVo, HCT116, HCT8, SW480, SW620, HT29, CaCO_2_, and RKO were purchased from American Type Culture Collection (ATCC, USA). All cells were cultured in a DMEM medium (BioInd, Beit Haemek, Israel) supplemented with 10% fetal bovine serum (BioInd) in 5% CO_2_ at 37 °C.

### Transfection

The siRNA of INHBA (siRNA1, siRNA2, and siRNA3) were purchased from RiboBio (Guangzhou, China). The plasmid encoding human INHBA was constructed by PCR amplification and subcloned into pEGFP-C1 expression vector to obtain pEGFP-INHBA. Following the manufacturer’s instructions, the transfection was carried out with a jetPRIME kit (Polyplus Transfection, Illkirch, France). LoVo cells were transfected with INHBA siRNA or negative control siRNA. pEGFP-INHBA or control vector (vec) were transfected into CACO2 and SW480 cells.

### Quantitative real‐time PCR (qRT-PCR)

Trizol reagent (Invitrogen, Thermo Fisher Scientific, Waltham, MA) was applied to isolate the total RNA. Reverse transcription reactions were conducted with the GoScript Reverse Transcription System (Promega, Madison, WI). Quantitative real-time PCR was implemented as previously described [23]. The 2^−ΔΔCt^ method was used to measure the expression of INHAB and GAPDH (endogenous control). The sequences of all the primer sets used in this study are listed in Supplementary Table S1.

### Western blotting

The detailed procedure of WB was performed according to the previous research [24]. The primary antibodies applied for immunoblotting included INHBA (Proteintech, China), SMAD2 (ZENBIO, Chengdu, China), Phospho-Smad2-S465/S467 (ABclonal), PI3 Kinase p85 (Cell Signaling Technology, Danvers, MA, USA), Phospho-PI3 Kinase p85 (Cell Signaling Technology), AKT (Cell Signaling Technology), Phospho-Akt (Ser473) (Cell Signaling Technology), GFP (Abcam, Cambridge, MA, USA), and (Utibody, Tianjin, China). Secondary antibodies used in this study were purchased from Proteintech (Wuhan, China). ECL reagents (Cwbiotech, Beijing, China) were used to detect the signals. The original images of all western blots were displayed in Supplementary Fig. S5.

### Cell proliferation assays

Cell Counting Kit-8 (CCK-8), 5-Ethynyl-2’-deoxyuridine (EdU) (RiboBio, Guangzhou, China), and colony formation assays were performed in this study to evaluate cell proliferation ability. After seeding cell suspension in 96-well culture plates (5000 cells/well), the cells were treated with CCK-8 (10 µL/well) for 2 h, and the optical density value was detected at 450 nm. For the EdU assay, cells were seeded into 96-well plates (1 × 10^5^ cells/well) and incubated in 5% CO_2_ at 37 °C for 24 h. According to the manufacturer’s protocol, cells were treated with EdU solution (100 µL/well) for 2 h, then stained with Apollo and Hoechest33342 after being fixed with 4% paraformaldehyde. EdU intensity was calculated using ImageJ (National Institutes of Health, USA).

### Cell cycle and apoptosis assay

After starvation for 24 h, cells were treated with metformin for 24 h or transfected with the INHBA OE plasmid or siRNA for 48 h. We collected the cells and fixed them with ice-cold 70% ethanol for 4 °C overnight, and stored them at −20 °C until analysis. The cells were then dyed with PI/RNase Staining Buffer (BD Biosciences, NJ, USA) and assessed by a fluorescence-activated cell sorting (FACS) Calibur system (BD Biosciences). The Annexin V assay was performed using FITC Annexin V/Dead Cell Apoptosis kit (BD Biosciences, NJ, USA). CRC cells were seeded in cell culture dishes at a density of 5 × 10^5^ cells, incubated for 24 h, and subsequently treated with metformin or PBS for 24 h. The cells were collected and suspended in 1× annexin-binding buffer and stained with FITC and PI for 15 min at room temperature in the dark. the stained cells were analyzed by a FACS Calibur system (BD Biosciences).

### ELISA assay

The secretion of INHBA (mlBio, Shanghai, China) and activin A (Abcam, Cambridge, MA) was measured with the indicated ELISA kit according to the instructions. The OD value was read at 450 nm.

### Immunohistochemistry (IHC)

We performed IHC staining as previously described [25]. IHC scores were based on the staining intensity in CRC tissues and the percentage of positively stained cells. INHBA staining was assessed at high (400×) magnification in five fields. The intensity scores were divided into four grades, including 0 (negative), 1 (weak), 2 (moderate), and 3 (strong). Scores of the percentage of positively stained cells were 1 (0–25%), 2 (26–50%), 3 (51–75%), and 4 (>75%). Staining percentage × intensity formed the comprehensive scores. The expression level of INHBA was finally divided into low [0–6) and high [6–12] groups according to the comprehensive scores.

### Reagents and chemicals

Metformin was purchased from Sigma-Aldrich (St. Louis, MO, USA), and dissolved in sterile PBS. The final concentration of metformin used in this study was 10 mM. SB431542 was purchased from MedChemExpress (New Jersey, USA), and dissolved in dimethyl sulfoxide (DMSO). The final concentration of SB431542 used in this study was 10 μM.

### Bioinformatic analysis

Microarray data of CRC samples from GSE67342 and GSE15781 data sets were collected from the GEO database. DEGs with significant differences were screened out according to the criteria of | log FC | > 2 and *p* < 0.05. The DEGs with significant differences in GSE67342 (24 h metformin treatment vs. 24 h control) and GSE15781 (tumor vs. normal) were analyzed by GEO2R. Molecular signatures of INHBA expression enrichment were analyzed by GSEA. Using the GEPIA website (http://gepia.cancer-pku.cn/), we analyzed the mRNA level of INHBA in CRC tissues and normal colon tissues and the correlation between the expression of INHBA and the overall survival of CRC patients in the TCGA. String database (https://www.string-db.org/) and GEPIA database were applied to evaluate the links between INHBA and TGF-β pathway. Cox proportional hazard regression model was conducted to divided samples into INHBA low expression group and INHBA high expression group. We draw Kaplan-Meier plots for survival analysis and utilized the log-rank test for statistical significance calculation.

### Statistical analysis

All the experimental data were analyzed by GraphPad Prism 8.0 (GraphPad Software Inc.) and SPSS software (version 16.0). The expression of INHBA in CRC tissues and normal tissues was compared using a paired *t* test and student *t* test. The significance comparison between groups was assessed by one-way ANOVA or Student’s *t* test. Log-rank test was applied to assess survival rates. The results were reported as the mean ± SD. *P* < 0.05 was considered statistically significant (*P < 0.05, **P < 0.01, ***P < 0.001).

## Results

### Metformin strongly inhibited the proliferation of CRC cells

**Fig. 1:**
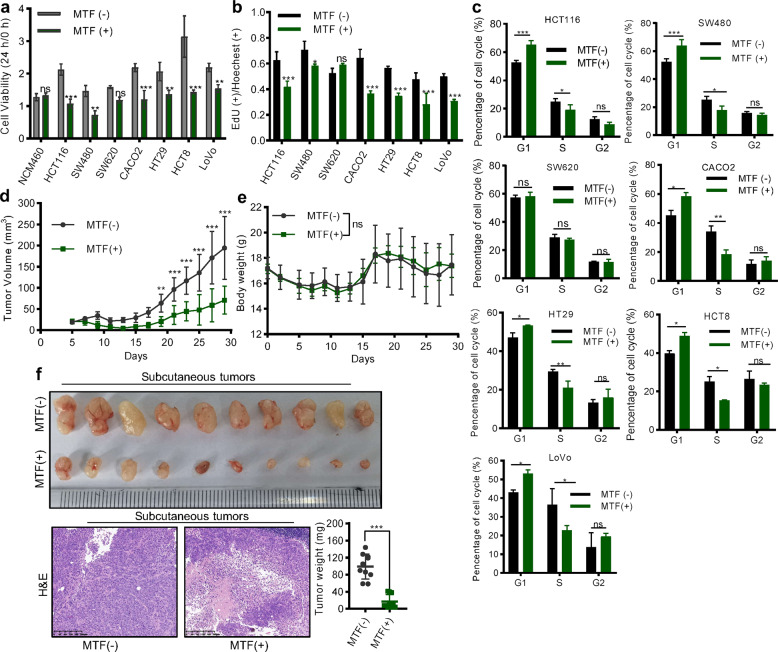
Metformin suppressed the proliferation of CRC. **a** CCK8 assay detection of the cell viability of NCM460 cell and 7 CRC cells with or without 10 mM metformin treatment (Student’s *t* test). MTF metformin. **b** EdU assay evaluated the percentage of proliferative cells after treating 10 mM metformin for 24 h (Student’s *t* test). **c** Flow cytometry showing the effect of metformin on cell cycle distribution (Student’s *t* test). **d**, **e** The growth rate of the subcutaneous tumors (**d**) and the bodyweight of mice (**e**) with or without metformin treatment (two-way ANOVA). **f** The image, weight, and representative HE staining of subcutaneous tumors. Magnification: 50×. Scale bar: 200 µm. The values are shown as the mean ± SD and experiments were independently repeated three times. *P < 0.05, ***P < 0.001.

We treated the CRC cells with 10 mM metformin to investigate its function and found that metformin effectively reduced the cell viability of most CRC cell lines while having little effect on the viability of normal colon cells (NCM460) (Fig. 1a). Further, we demonstrated that metformin treatment for 24 h suppressed the proliferation of CRC cells using EdU staining (Fig. 1b, Supplementary Fig. S1a). However, treating with 10 mM metformin for 24 h had no significant effect on the apoptosis of SW480, LoVo, and HCT8 cells (Supplementary Fig. S1b). Thus, we speculated that short-term metformin treatment may mainly affect the proliferative phenotype of CRC cells. Meanwhile, we confirmed metformin caused the inhibition of cell growth by arresting the cell cycle in the G1 phase (Fig. 1c). We also constructed a subcutaneous xenograft model of CRC and found metformin treatment greatly inhibited tumor growth, reduced tumor weight, and had a little toxic side effect (Fig. 1d-f). The results above suggested that metformin has an anti-cancer effect in CRC by suppressing tumor proliferation.

### Identifying the potential targets of metformin in CRC

**Fig. 2:**
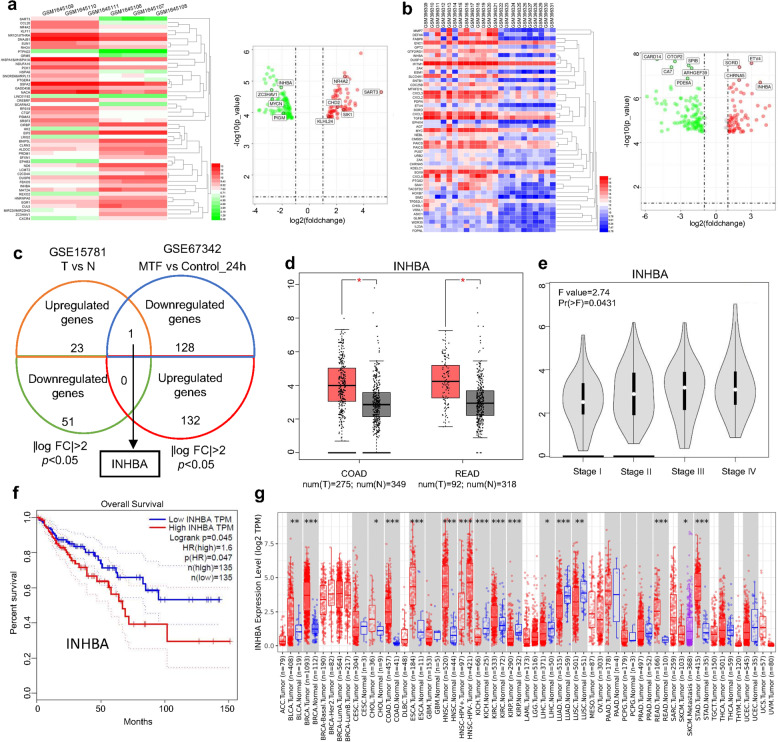
Screening target gene of metformin in CRC. **a** Analysis of the differential genes (DEGs) between metformin-treated group (24 h) and control group (24 h) in GSE67342 dataset. **b** Heatmap and volcano map showing DEGs between CRC tissues and adjacent tissues in GSE15781 profile. **c** Comparison of the genes altered in GSE67342 (|log2FC | >2; *p* < 0.05) with GSE15781 dataset (|log2FC | >2; *p* < 0.05). **d**, **e** GEPIA online database analyzing the mRNA expression of INHBA in CRC tissues and normal colon tissues (**d**), as well as the expression of INHBA in CRC tissues from the different stages (**e**). **f** Kaplan–Meier curve showing the OS probability of CRC patients from the GEPIA database with low or high mRNA levels of INHBA (log-rank test). OS overall survival. **g** Expression of INHBA in various tumor tissues and their adjacent tissues. Red boxplot: tumor tissues; Bule boxplot: normal tissues; Purple boxplot: metastatic tissues. The data was gained from the TIMER database.

Since we verified the antitumor effect of metformin in CRC, we set out to screen for potential target genes that mediated the effects of metformin. We initially obtained a representative dataset from Gene Expression Omnibus (GEO) database. We analyzed the differential gene expression (DEGs) between LoVo cells treated with metformin for 24 h and control cells in GSE67342 microarray [26], and found 132 upregulating genes and 129 downregulating genes (|log FC | > 2, *p* < 0.05) (Fig. 2a). Through performing the GO analysis of the significantly altered gene sets in GSE67342 (24 h treatment group), we found those genes mainly enriched in the G1/S transition of the mitotic cell cycle (Supplementary Fig. S2). Additionally, analyzing the gene expression in colon cancer tissues and normal tissues in the GSE15781 dataset [27], we screened a set of genes with the standard of |log FC | > 2 and *p* < 0.05, which may be involved in CRC progression (Fig. 2b). Comparison with significantly altered genes in GSE67342 (24 h metformin treatment group) with GSE15781(tumor vs. normal) dataset showed that only INHBA was highly expressed in CRC tumor tissue and simultaneously downregulated after metformin treatment (Fig. 2c). The result suggested that INHBA may be the most likely target of metformin in CRC. Therefore, we utilized TCGA databases to evaluate the expression of INHBA in CRC samples and its impact on the prognosis of patients. The results showed that the mRNA expression of *INHBA* was consistently higher in tumor tissues than those adjacent tissues and the statistical analysis showed great significance (Fig. 2d). Moreover, the tumor tissues from the advanced stage represented a much higher level of INHBA than those for early stage (Fig. 2e). Thus, the patients with a higher level of INHBA showed a shorter overall survival time (Fig. 2f). It is worth mentioning that in addition to CRC, INHBA is highly expressed in various tumor tissues, such as breast cancer and esophageal squamous cell carcinoma (Fig. 2g). Based on the results above, we considered INHBA as the most possible target gene of metformin for its antitumor function.

### Metformin reduced the expression of INHBA in CRC

**Fig. 3:**
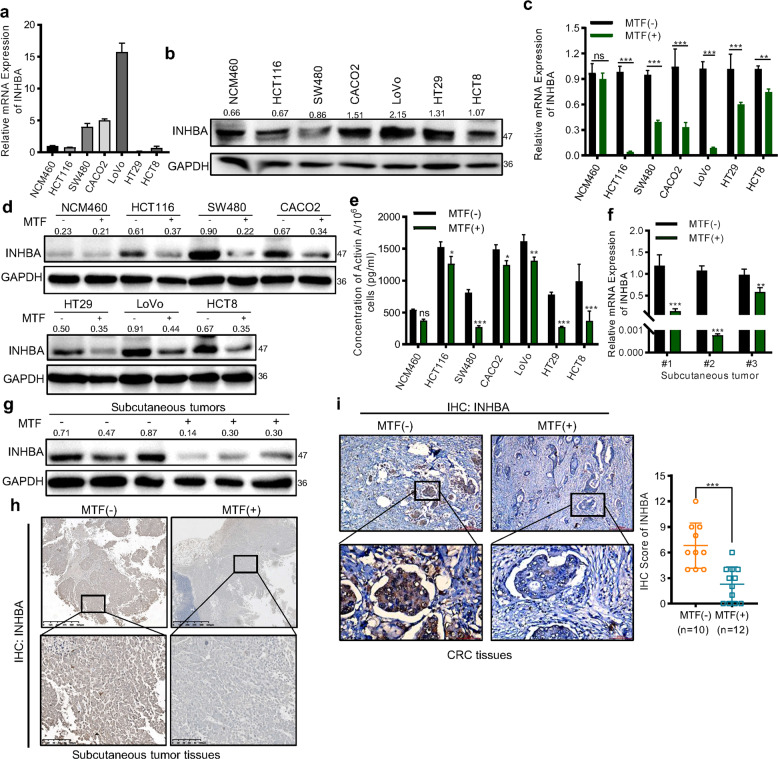
Metformin inhibited the expression of INHBA in CRC. **a**, **b** qPCR (**a**) and western blot (**b**) detecting the mRNA and protein level of INHBA in NCM460 and CRC cells. **c**, **d** qPCR (**c**), and western blot (**d**) testing the effect of metformin (10 mM, 24 h treatment) on the expression of INHBA. **e** ELISA measured the secretion of activinA after metformin treatment. **f**–**h** qPCR (**f**), western blot (**g**), and IHC (**h**) evaluating the expression change of INHBA in subcutaneous tumors from control and metformin treatment groups. **i** Representative IHC images of CRC tissues with or without metformin treatment stained with an INHBA antibody (*n* = 22). Up: Magnification: 50×; Scale bar: 200 µm. Low: Magnification: 200×; Scale bar: 50 µm. The values are shown as the mean ± SD and experiments were independently repeated three times. *P < 0.05, ***P < 0.001. The data in this figure were analyzed by Student’s *t* test.

To confirm the target relationship between metformin and INHBA, we firstly detected the mRNA and protein level of INHBA in six CRC cell lines and NCM460 cells (Fig. 3a, b, Supplementary Fig. S3). Then, treating cells with 10 mM metformin for 24 h to measure the change of mRNA and protein expression, we found that both the mRNA and protein expression of INHBA in CRC cells rather than NCM460 cells were significantly downregulated by metformin (Fig. 3c, d). As the consequence, the secretion of activinA was reduced after metformin treatment (Fig. 3e). Consistently, relative to the control group, the expression of INHBA in xenograft tumors was significantly decreased in the metformin group (Fig. 3f–h). Moreover, we collected paraffin samples of tumor tissues from 22 diabetic patients with CRC and subjected them to immunohistochemistry (IHC) staining. Among those samples, we found the staining intensity of INHBA in tumor tissues of patients who received metformin treatment was weaker than that of patients treated with other hypoglycemic drugs (Fig. 3i). The results suggested that metformin could effectively downregulate INHBA expression in CRC.

### Downregulation of INHBA achieved the similar anti-tumor effect as metformin treatment in CRC cells

**Fig. 4:**
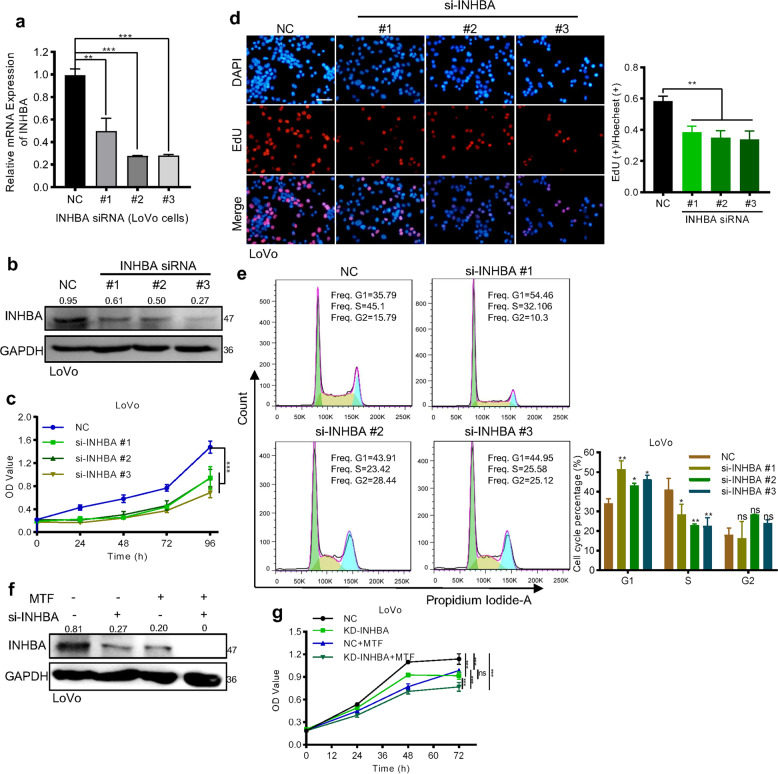
Silencing INHBA inhibited the growth of CRC cells. **a**, **b** qPCR (**a**) and western blot (**b**) were used to verify the knockdown efficiency of INHBA (Student’s *t* test). **c**, **d** CCK8 (**c**), and EdU assay (**d**) analyzed the proliferation of LoVo cells after knocking down INHBA (Student’s *t* test). **e** Flow cytometry was used to measure cell cycle distribution. **f** Western blot detecting a synergistic effect of metformin and siRNA in downregulating the expression of INHBA. **g** CCK8 showing the synergistic effect of metformin and siRNA in the proliferation of LoVo cells. The values are shown as the mean ± SD and experiments were independently repeated three times. *P < 0.05, ***P < 0.001. The data in this figure were analyzed by two-way ANOVA.

Next, we want to understand the role of INHBA on CRC proliferation. We specifically silenced INHBA in LoVo cells with three different siRNA (Fig. 4a, b). CCK8 and colony formation assay showed that silencing INHBA greatly inhibited the growth rate of LoVo cells (Fig. 4c, Supplementary Fig. S4). Consistently, the EdU and flow cytometry assay revealed that knocking down INHBA mainly reduced the tumor cell proliferation by causing G1/S arrest (Fig. 4d, e). Additionally, when we treated LoVo cells with INHBA siRNA, metformin, respectively or combination themselves, we found that siRNA or metformin treatment on LoVo cells achieved a similar inhibition effect on the protein level of INHBA, while the combination treatment showed a synergistic effect on the downregulation of INHBA (Fig. 4f). Consistently, both siRNA and metformin treatment suppressed the proliferation of LoVo cells and there was no difference in inhibition rate between the two groups (Fig. 4g). As expected, due to dual inhibition of INHBA expression, the siRNA of INHBA and metformin showed a synergistic effect in inhibiting tumor proliferation (Fig. 4g). All of the above results provide sufficient evidence that the anti-tumor effect of metformin in CRC is realized by downregulating INHBA.

### Overexpression of INHBA partially resisted the anti-proliferation effect of metformin

**Fig. 5:**
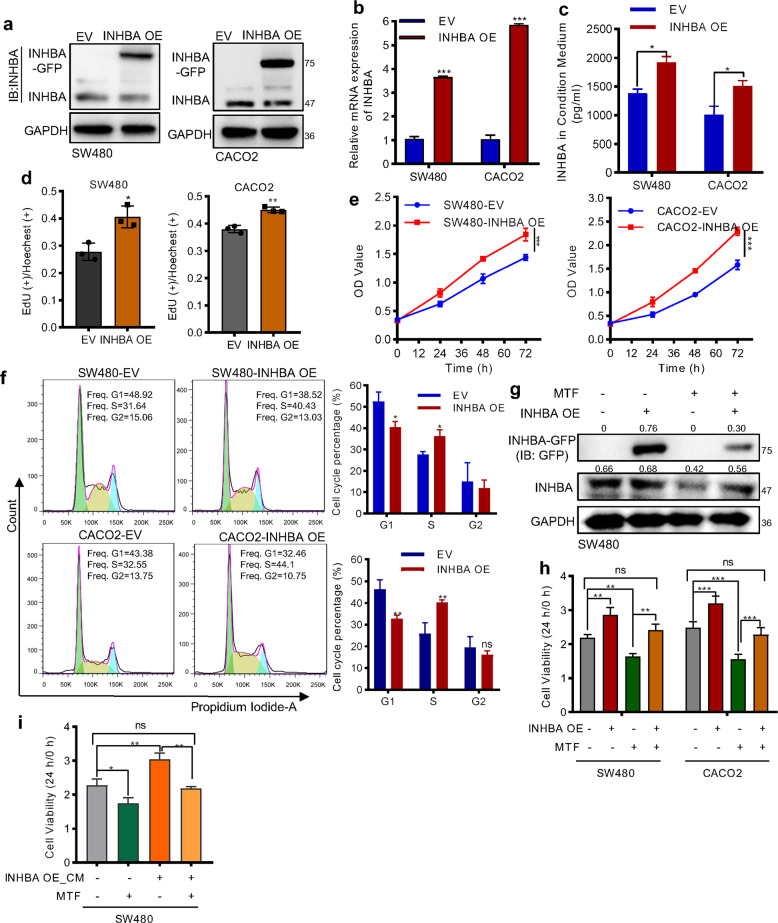
Overexpression of INHBA antagonized the antitumor effect of metformin. **a**, **b** Western blot (**a**), and qPCR (**b**) was used to confirm the efficiency of INHBA overexpression. **c** The concentration of INHBA in a conditioned medium of 1 × 10^6^ cells was determined by ELISA. **d**–**f** EdU (**d**), CCK8 (**e**), and flow cytometry (**f**) showing the promotion effect of INHBA on tumor cell growth. **g** Western blot analyzing the protein level of INHBA. INHBA-GFP: the exogenous fusion protein. **h** CCK8 analyzing the effect of metformin on cell viability after overexpression INHBA. **i** CCK8 detecting the influence of condition medium from the INHBA overexpression cells on metformin inhibiting SW480 cell growth. CM: condition medium from the cells overexpressing INHBA. The values are shown as the mean ± SD and experiments were independently repeated three times. *P < 0.05, ***P < 0.001. The data in this figure were analyzed by two-way ANOVA.

Since silencing INHBA inhibited the proliferation of CRC cells, we wanted to explore whether overexpression of INHBA can resist the effect of metformin. We constructed the INHBA overexpression vector and transfected the plasmid to SW480 and CACO_2_ cells. The qRT-PCR and western blot results confirmed the successful expression and secretion of exogenous INHBA in CRC cells (Fig. 5a–c). Further, proliferation assays indicated that overexpression of INHBA evidently promoted the growth rate of SW480 and CACO2 cells by accelerating G1/S transition (Fig. 5d–f). Then, we treated SW480 cells with INHBA overexpression plasmid, metformin, individually or combined themselves together for protein expression and functional tests. The results revealed that exogenous overexpression of INHBA in metformin-treated cells partially restored the level of INHBA and the anti-proliferation effect of metformin (Fig. 5g, h), Meanwhile, we collected the conditioned medium (CM) from the cells stable overexpressing INHBA, and treated SW480 cells with metformin and CM to observe the change of cell viability. The result showed that the CM from INHBA overexpression cells partly rescued the cell proliferation ability which was inhibited by metformin treatment (Fig. 5i). Altogether, these data demonstrated that the therapeutic effect mediated by metformin is attenuated in CRC cells with high expression of INHBA.

### Metformin blocked the activation of TGF-β signaling by suppressing INHBA

**Fig. 6:**
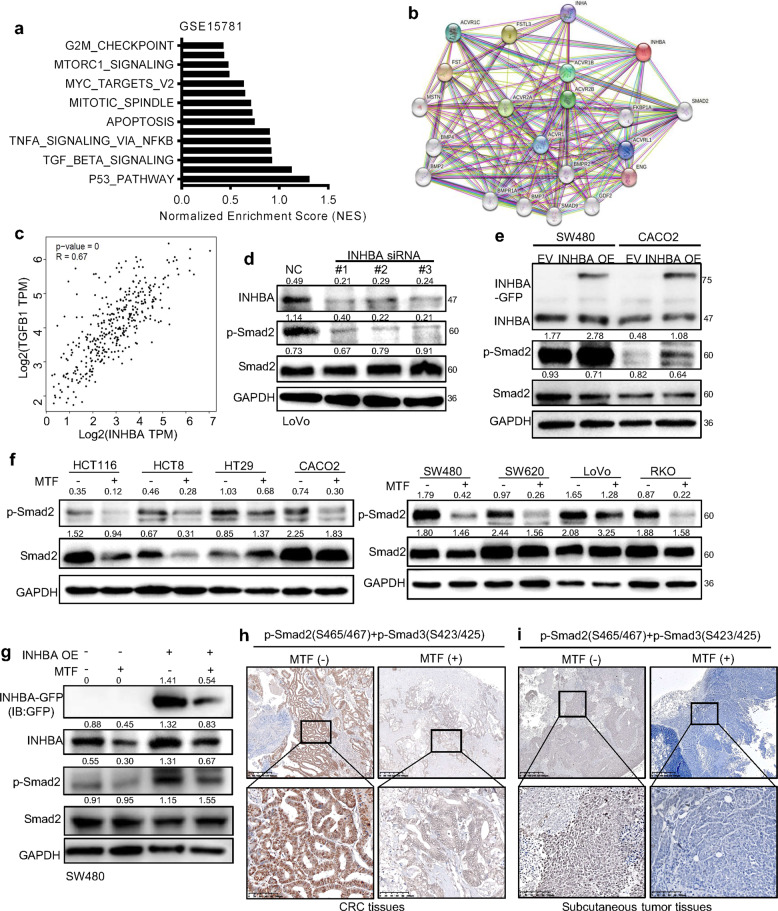
Metformin attenuated the activity of TGF-β signaling via downregulating INHBA. **a** GSEA analyzed INHBA positive-associated gene sets in the GSE15781 dataset. **b** Prediction of proteins interacting with INHBA by STRING database. **c** The correlation of INHBA and TGF-B1 in CRC tissues. Data from GEPIA database. **d**, **e** Knocking down (**d**), or overexpressing (**e**) INHBA decreased or increased the activity of TGF-β pathway. **f** Metformin treatment reduced the phosphorylation level of Smad2 in various CRC cells. **g** Exogenous expressing INHBA partly rescued the inhibition effect of metformin on the activity of TGF-β/smad signaling. **h**, **i** A represent image of IHC showing metformin treatment inactivated TGF-β signaling in CRC tissues (**h**) and subcutaneous tumors (**i**). Magnification: 100× (low); 40× (up). Scale bar: 100 µm (low); 400 µm (up). The values are shown as the mean ± SD and experiments were independently repeated three times. *P < 0.05, ***P < 0.001.

Although we confirmed INHBA is an important target for metformin in CRC, we still wondered which signaling pathway is responsible for the INHBA mediated malignant promoting phenotypes in CRC. We performed the gene set enrichment analysis (GSEA) to determine general functional features of INHBA positive-associated genes in the GSE15781 dataset (Fig. 6a). Combined with the information of proteins interacting with INHBA in the string database (Fig. 6b), the results strongly suggested that INHBA may be involved in the activation of the TGF-β signaling pathway. Meanwhile, applying the GEPIA database, a highly positive correlation between the mRNA expression of INHBA and TGFB1 was found in CRC (Fig. 6c). To validate the data acquired from the public database, we detected the activation status of the TGF-β signaling pathway after silencing and overexpressing INHBA in CRC cells, respectively. Knocking down INHBA in LoVo cells greatly reduced the phosphorylation of Smad2, and overexpressing INHBA achieved the opposite result (Fig. 6d, e). The results above suggest that INHBA is one of the activators of the TGF-β signaling pathway. To investigate whether metformin is capable to regulate the activity of TGF-β signaling through targeting INHBA, we treated CRC cells with metformin and found the phosphorylation level of Smad2 was decreased in all cells receiving metformin treatment (Fig. 6f). Moreover, overexpression of INHBA reversed the inhibitory effect of metformin on the TGF-β signaling pathway to a certain extent (Fig. 6g). Further, we conducted IHC to measure the activation state of TGF-β signaling in CRC tissues and xenograft tumor tissues from the metformin treatment group and control group. The results showed that the staining strength of pSmad2/3 in the metformin treatment group was much weaker than that of the control group (Fig. 6h-i). The IHC results prompted that metformin treatment could reduce the activity of TGF-β signaling in tumor tissues. Taking together, the anti-tumor effect of metformin in CRC depends on the inhibition of the TGF-β pathway.

### Metformin indirectly affected PI3K/Akt via inactivation of TGF-β signaling

**Fig. 7:**
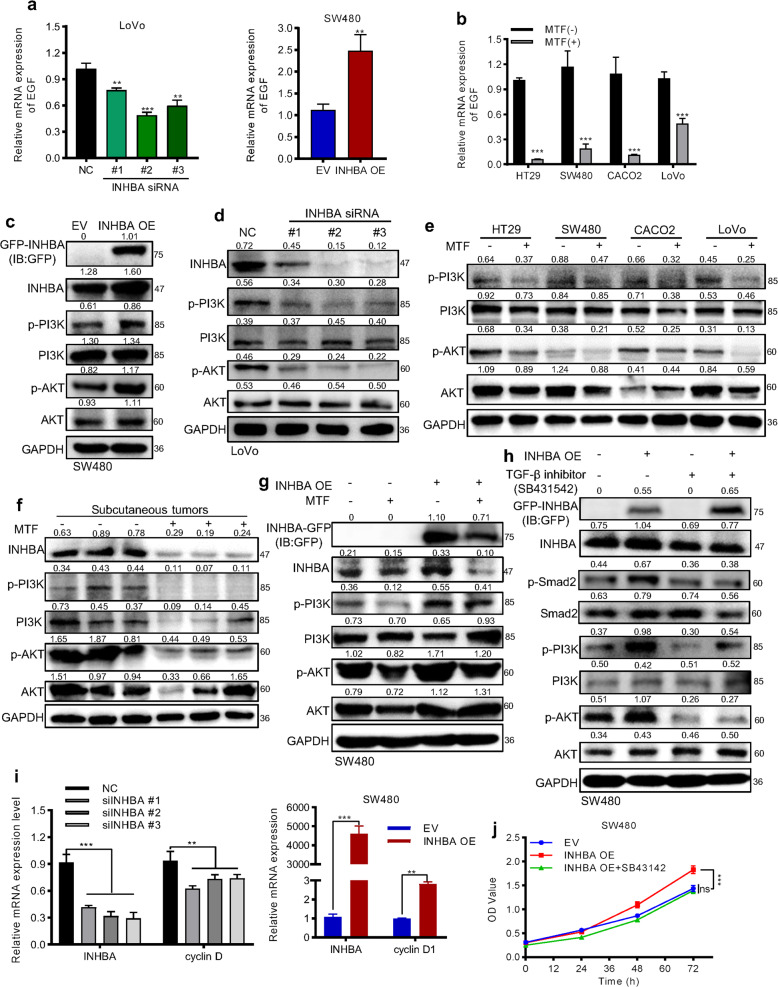
Metformin indirectly inhibited the activation of the PI3K/AKT pathway to suppress cell proliferation. **a**, **b** qPCR measured the mRNA expression of EGF after altering INHBA (**a**) or treating with metformin (**b**). **c**, **d** upregulating (**c**) or silencing (**d**) INHBA enhanced or mitigated the activity of the PI3K/AKT pathway. **e**, **f** Metformin treatment restraint of PI3K/AKT activation in CRC cells (**e**) and subcutaneous tumor tissues (**f**). **g** PI3K/AKT signaling activity after INHBA overexpression and metformin treatment. **h** TGF-β inhibitor blocked the activation of PI3K/AKT signaling pathway induced by INHBA overexpression. SB431542, an inhibitor of activinR I and TGFβR I. **i** INHBA positively affected the mRNA level of cyclinD1 (Student’s *t* test). **j** TGF-β inhibitor counteracted the acceleration of cell proliferation induced by INHBA overexpression. The values are shown as the mean ± SD and experiments were independently repeated three times. *P < 0.05, ***P < 0.001.

In tumors, activation of TGF-β signaling has two opposite effects on cell proliferation, and its cell growth promotion effect always depends on transcriptionally upregulating EGF to indirectly activate PI3K/AKT pathway [28]. Through qPCR assay, we found the expression of EGF showed positively changed very well following the down or up-regulation of INHBA (Fig. 7a). Correspondingly, metformin decreased the mRNA level of EGF in CRC cells (Fig. 7b). Further, western blot results showed overexpression INHBA in SW480 cells resulted in activation of PI3K/AKT pathway (Fig. 7c). When knocking down INHBA or treating CRC cells with metformin, the activity of PI3K/AKT was greatly repressed (Fig. 7d, e). Also, the activity of PI3K/AKT signaling in xenograft tumors was reduced after treating metformin (Fig. 7f). Exogenous expression of INHBA in SW480 cells partly restored the inhibitory effect of metformin on PI3K/AKT signaling (Fig. 7g). In order to verify the activity of PI3K/AKT pathway in CRC is regulated by TGF-β signaling activation status, we treated cells with the inhibitor of TGF-β signaling (SB431542, inhibitor of activinRI and TGFβRI) and found the activity of PI3K/AKT was significantly reduced (Fig. 7h). Moreover, this inhibition effect on PI3K/AKT signaling cannot be rescued by INHBA overexpression (Fig. 7h). As a consequence, down or upregulation of INHBA decreased or increased the expression of CyclinD1 (Fig. 7i), and the cell growth promotion effect of INHBA can be blocked by the TGF-β pathway inhibitor (Fig. 7h). All results suggested that metformin performed its anti-proliferation function through blocking INHBA/ TGF-β/PI3K/AKT/cyclinD1 axis activation in CRC.

### INHBA was a potential biomarker for CRC prognosis

**Fig. 8:**
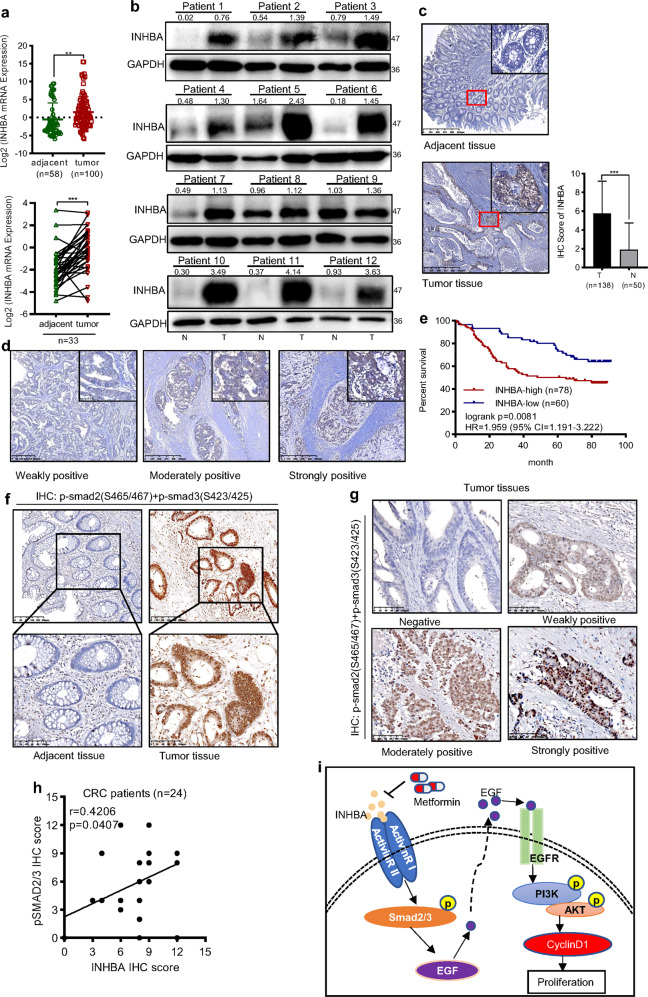
INHBA was highly expressed in human CRC tissues and correlated with unfavorable prognosis. **a** qPCR analysis of the mRNA levels of INHBA among CRC and adjacent normal tissues (Student’s *t* test), as well as 33 pairs of tumor tissues and adjacent normal tissues (paired *t* test) (low). **b** INHBA protein levels in fresh CRC (T) and adjacent normal samples (N) (*n* = 12) were detected by western blotting. **c** Representative IHC images of CRC tissues and corresponding normal tissues stained with an INHBA antibody (*n*(*T*) = 138, *n*(*N*) = 50) (Student’s *t* test). Magnification: 200× (small); 40× (large). Scale bar: 50 µm (small); 400 µm (large). **d** The representative immunohistochemical images showing different staining intensities of INHBA in CRC tissues. Magnification: 200× (small); 40× (large). Scale bar: 50 µm (small); 400 µm (large). **e** Kaplan–Meier curve showing the OS of patients with CRC according to INHBA expression (log-rank test). **f** Representative IHC images of p-smad2(S465/467) and p-smad3(S423/425) staining in CRC tissues and paracancerous tissues. **g** Representative images showing the staining strength of p-smad2/3 in CRC tissues. Magnification: 100×. Scale bar: 100 µm. **h** The correlation between INHBA expression and p-smad2/3 expression in 22 CRC tissues. **i** Schematic description of the main molecular mechanism of metformin suppressing CRC proliferation. OS overall survival, HR hazard ratio, CI confidence interval. The values are shown as the mean ± SD and experiments were independently repeated three times.

Finally, we evaluated the expression and the potential roles of INHBA in clinical samples of CRC. We collected 100 CRC tumor tissues and 58 paracancerous tissues to identify the expression level of INHBA. The results showed that the mRNA of *INHBA* was significantly upregulated in CRC tissues compared with adjacent normal tissues, and the RT-qPCR analysis of 33 paired clinical CRC samples showed similar results (Fig. 8a). Consistent with the change of mRNA level, the protein expression of INHBA in CRC tissues from 12 patients was also markedly higher than that of normal tissues (Fig. 8b). Moreover, we observed the staining intensity of INHBA in CRC tissues was stronger than that in paracancerous tissues, and it was mainly located in the cytoplasm (Fig. 8c, d). After confirming the high expression of INHBA in CRC, we further analyzed the influence of INHBA expression on the survival time of patients. The Kaplan–Meier survival analysis showed that the overall survival time of CRC patients with high INHBA expression was shorter than those with INHBA low expression (Fig. 8e). Meanwhile, the TGF-β signaling was activated in CRC tissues and protein levels of INHBA and pSMAD2/3 showed a positive correlation (Fig. 8f–h). Next, we investigated the association between INHBA levels and clinicopathological characteristics of CRC patients, and the tissue samples were divided into low (*n* = 60) and high (*n* = 78) INHBA expression groups. The results indicated that the expression of INHBA protein was correlated with the histological differentiation (*p* = 0.0434), TNM stage (*p* = 0.0053), lymph node metastasis (*p* = 0.0262) and overall survival (*p* = 0.0383) (Table 1). Collectively, these results suggest that the high expression of INHBA is closely related to poor prognosis and can be used as a potential biomarker for CRC cancer.

**Table 1:** Association between INHBA levels in tumor tissues and clinicopathological characteristics of CRC patients.

Variable	INHBA expression low (*N* = 60) high (*N* = 78)		^A^*P* value
*Sex, N (%)*			0.4863
M	39 (45.88)	46 (54.12)	
F	21 (39.62)	32 (60.38)	
*Age at diagnosis, N (%)*			0.4818
≧60	21 (38.89)	33 (61.11)	
<60	39 (46.43)	45 (53.57)	
*Histological differentiation, N (%)*			**0.0434**
Well	24 (54.54)	20 (45.45)	
Moderate	28 (45.16)	34 (54.84)	
Poor	8 (25.00)	24 (75.00)	
*TNM Stage, N (%)*			**0.0053**
I–II	34 (57.63)	25 (42.37)	
III–IV	26 (32.91)	53 (67.09)	
*Lymph node metastasis, N (%)*			**0.0262**
Absent	40 (51.95)	37 (48.05)	
Present	20 (32.79)	41 (67.21)	
*Distant metastasis, N (%)*			0.2991
Absent	38 (47.5)	42 (52.5)	
Present	22 (37.93)	36 (62.07)	
*Overall survival, N (%)*			**0.0383**
Alive	39 (52.00)	36 (48.00)	
Dead	21 (33.33)	42 (66.67)	

Samples: The Xiangya Hospital (Changsha, China); ^A^*χ*^2^ test; *n* = 138; bold values indicate statistical significance, *p* < 0.05.

## Discussion

As an emerging potential anticancer agent, metformin has arrested much attention in CRC research. However, the exact molecular mechanisms for the anti-proliferative effect of metformin remain largely obscure. In the present study, we first discovered that INHBA forms the main target for metformin to inhibit proliferation in CRC. Furthermore, we also preliminarily clarified the molecular mechanism by which metformin suppresses the growth of CRC.

In recent years, increasing clinical investigations have shown that metformin significantly reduced various cancers risk, including CRC, pancreatic cancer, breast cancer, lung cancer, and prostate cancer [12, 29, 30]. It is well-known that sustaining chronic proliferation is the most fundamental trait of tumorigenesis [31], while the role of metformin in cancer therapy is largely attributed to its ability in inhibiting these hyperproliferative processes. Yang et al. [32] showed that metformin dramatically reduced the proliferation of HT-29 and HCT-116 cells in a concentration-dependent manner. Another study observed the prolongation of the cell cycle G1 phase in CRC cells with metformin treatment [33]. Consistent with the aforementioned research, we also confirmed metformin has a strong inhibitory capability on CRC cells growth by arresting the cell cycle in the G1 phase. Notably, through bioinformatic and statistical analysis, one of our novel discoveries is the significant down-regulation of INHBA in metformin-treated CRC cells, thus we speculate that the anti-proliferation effect of metformin in CRC may be mediated by INHBA.

INHBA is mainly involved in the formation of activin A, a disulfide-linked homodimer, initially identified for its ability to promote the release of follicle-stimulating hormone [34]. Subsequent studies have revealed multiple functions of INHBA, including the induction of embryonic stem cell differentiation [35] and vertebrate regeneration [36]. Over the last couple of decades, the role of INHBA in various malignant tumors has been gradually explored. It has been confirmed that INHBA was overexpressed in lung adenocarcinoma, which promoted tumor proliferation and correlated with poor outcomes in stage I disease [37]. As an indicator of poor prognosis, researchers found that the expression of INHBA was positively correlated with tumor diameter and infiltration in gastric cancer [38]. Furthermore, two previous studies demonstrated that the expression of INHBA is markedly increased in CRC clinical samples, which is a risk factor for poor prognosis in patients with CRC [39, 40]. In this study, we observed that INHBA was significantly upregulated in CRC cell lines and CRC tissues, and mainly located in the cytoplasm and can secret to CM. The expression of INHBA was closely related to tumor differentiation, TNM stage, lymph node metastasis, and overall survival in patients with CRC. All above suggests that INHBA may provide the patient a surveillance strategy to prevent CRC and treatment in case of incident CRC.

Importantly, our study is the first to link metformin and INHBA in cancer treatment. We found that metformin significantly reduced the expression of INHBA in both mRNA and protein levels. Previous findings have observed that INHBA downregulation led to diminutions in the invasion and proliferation of some cancer cells, such as prostate cancer stem cells [41] and gastric cancer cells [20]. The rate of DNA replication in ovarian cancer cells was also slowed by INHBA silencing [42]. Our in vitro experiments showed that silencing INHBA suppressed the growth of CRC cells by arresting the cell cycle in the G1 phase, whereas overexpression of INHBA resulted in an opposite effect. These results confirmed that the anti-proliferation effect of metformin in CRC cells is achieved by decreasing the expression of INHBA, and suggested that the expression of INHBA could predict the anticancer capability of metformin and could be applied as a screening index for metformin in CRC treatment. In addition, our data displayed that INHBA is highly expressed in a variety of tumor tissues, which provides a basis for the application of metformin in the treatment of other tumors by down-regulating INHBA. However, the detailed molecular mechanism of metformin downregulating INHBA is still unknown.

TGF-β signaling always plays a dual role in the progression of cancer. In the advanced stage of the tumor, activation of TGF-β signaling supports the proliferation and metastasis of the tumor [43]. TGF-β signaling includes Smad and non-Smad pathways. The cooperation between Smad and non-Smad pathways determines the final consequence of cellular reactions to TGF-β [44]. INHBA is a member of the TGF-β superfamily and is involved in the regulation of TGF-β activity. In INHBA-mediated fibroblast activation, Li et al. observed an increase in Smad2 phosphorylation [45]. Another research displayed that INHBA induced the malignant phenotype of CRC by triggering the activation of TGF-β signaling [22]. As a ligand of TGF-β signaling, INHBA simultaneously activated Smad and non-Smad pathways in TGF-β signaling to regulate the proliferation of CRC cells in our study. PI3K/AKT is classical TGF-β signaling of the non-Smad pathway [46]. However, the underlying molecular mechanisms of the TGF-β activating PI3K/AKT pathway are not well characterized. We noticed that pSmad2 transcriptionally upregulated the expression of EGF and activated PI3K/AKT signaling through EGF in liver cancer [28], and we confirmed this regulation relationship in CRC. Currently, the anticancer effects of metformin are mainly related to the activation of AMPK and the inhibition of mTORC1 and insulin/insulin-like growth factor-1 (IGF-1) signaling [47]. TGF-β/Smad signaling is a crucial pathway in the development of CRC, which promotes the growth and metastasis of tumors in the advanced stage [48]. Our work complements and summarizes the mechanism of the anti-proliferation effect of metformin in CRC and provides a possible mechanism for metformin to prevent CRC metastasis.

In summary, our study revealed the molecular mechanism of metformin in repressing the growth of CRC (Fig. 8i), providing more comprehensive guidance for the application of metformin in CRC therapy. Importantly, we have confirmed that INHBA is a critical target for the anti-proliferation role of metformin in CRC. Future studies could explore the therapeutic effect of metformin in other malignant tumors with high INHBA expression and the detailed regulation mechanism of metformin on INHBA expression.

## Supplementary information

supplementary figures and table

checklist

## Data Availability

All data generated or analyzed during this study are included in this published article.

## Material availability

All data generated or analyzed during this study are included in this published article.
