# Supplementary material for: Metformin suppresses the growth of colorectal cancer by targeting INHBA to inhibit TGF-β/PI3K/AKT signaling transduction
Source: Cell Death Dis. 2022 Mar 2;13(3):202. doi: 10.1038/s41419-022-04649-4 (PMC8891354; doi:10.1038/s41419-022-04649-4)
Supplement: Supplementary file 1 — supplementary figures and table [file 41419_2022_4649_MOESM1_ESM.pdf]

Figure S1

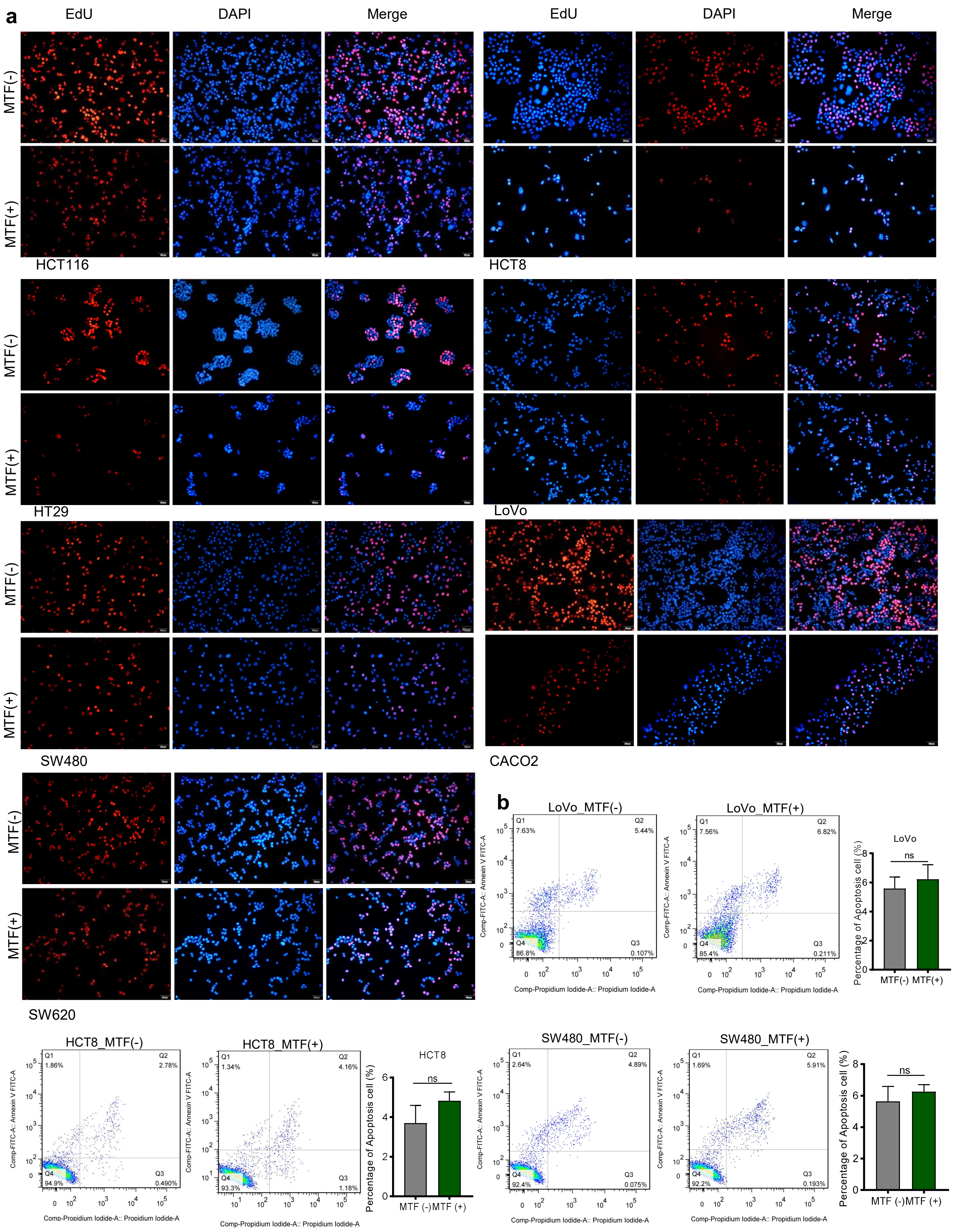

Figure S1 metformin suppressed proliferation of CRC cells. (a) Representative images of EdU staining in 7 CRC cell lines with or without metformin treatment. Magnification: 200×. Scale bar: 50 µm. (**b**) Flow cytometry showing the effect of low dose (10mM) metformin on cell apoptosis

Figure S2

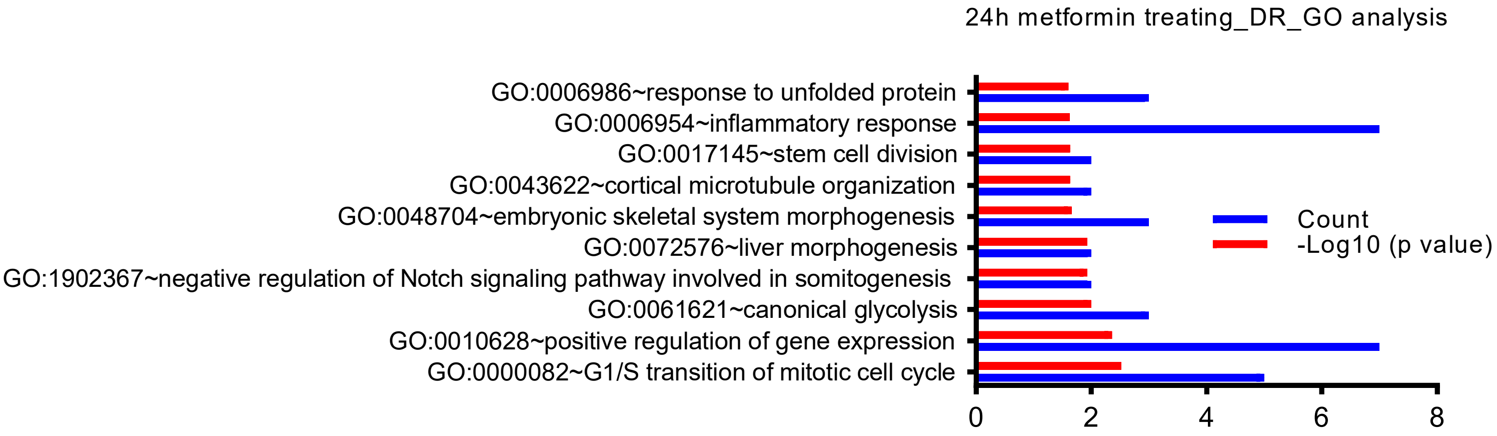

Figure S2 Metformin inhibited G1/S transduction in LoVo cells. GO analyzing the genes downregulated after metformin treating for 24 hours in GSE67342.

Figure S3

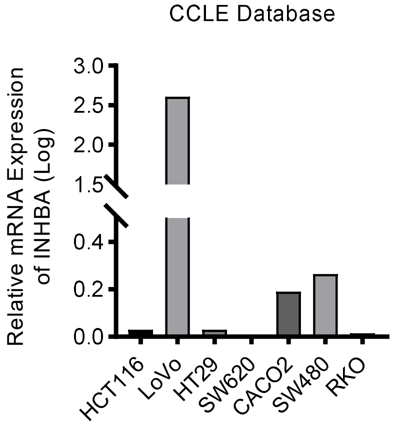

Figure S3 mRNA expression of INHBA in CRC cell lines. Quantitative PCR analysis of INHBA expressing in CRC cell lines. Data from Cancer Cell Line Encyclopedia (CCLE) database.

Figure S4

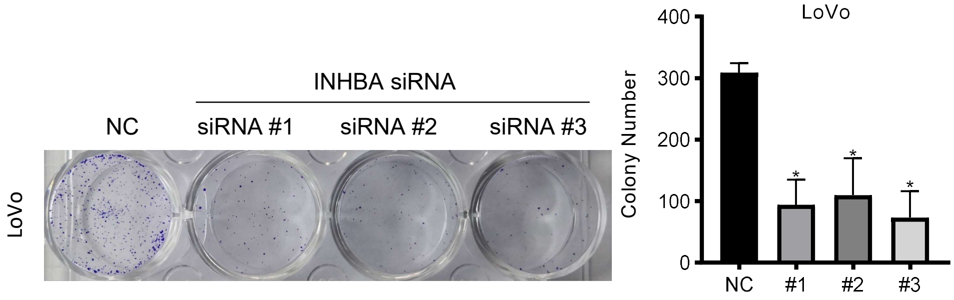

Figure S4 Knocking down INHBA reduced the viability of CRC cells. Colony formation assay showing silencing INHBA inhibited the growth of LoVo cells.

Figure S5

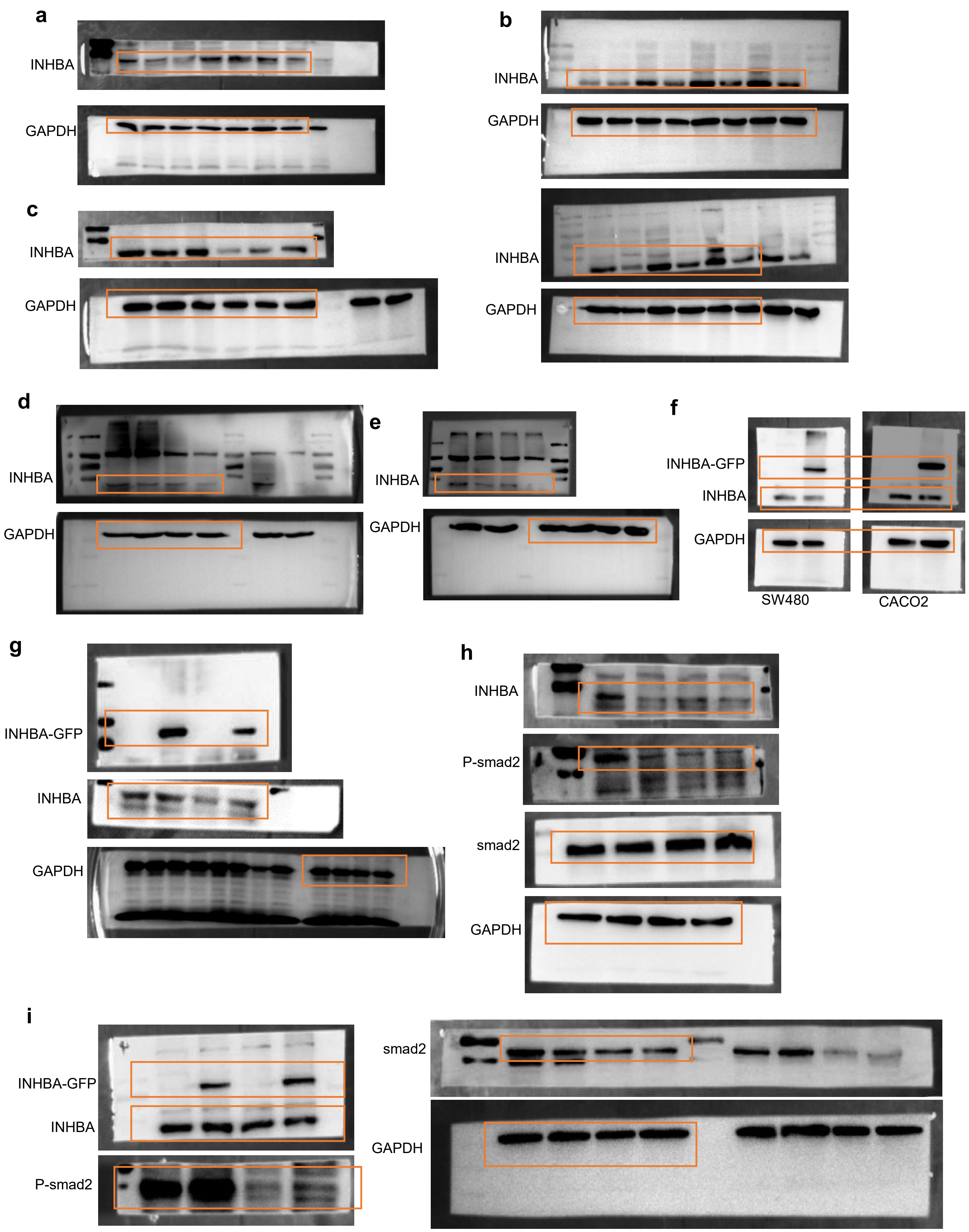
a
INHBA
GAPDH
b
INHBA
GAPDH
INHBA
GAPDH
c
INHBA
GAPDH
d
INHBA
GAPDH
e
INHBA
GAPDH
f
INHBA-GFP
INHBA
GAPDH
SW480
CACO2
g
INHBA-GFP
INHBA
GAPDH
h
INHBA
P-smad2
smad2
GAPDH
i
INHBA-GFP
INHBA
P-smad2
smad2
GAPDH

j

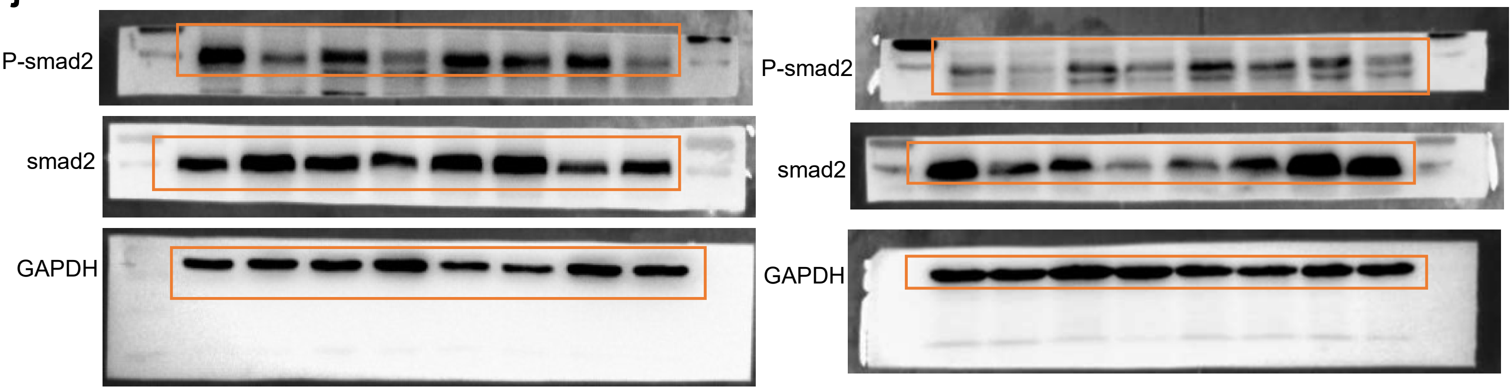
P-smad2
smad2
GAPDH
P-smad2
smad2
GAPDH

k

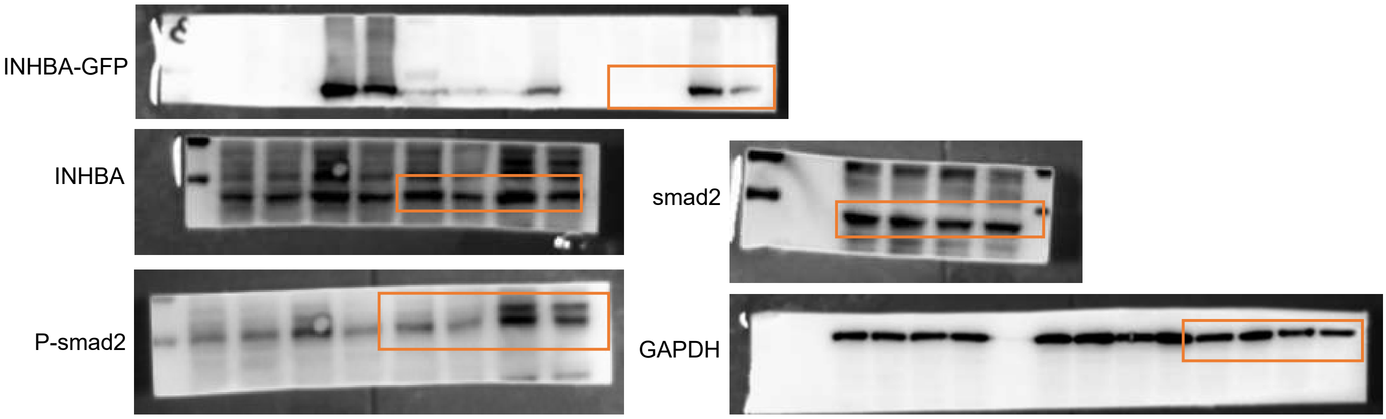
INHBA-GFP
INHBA
P-smad2
smad2
GAPDH

l

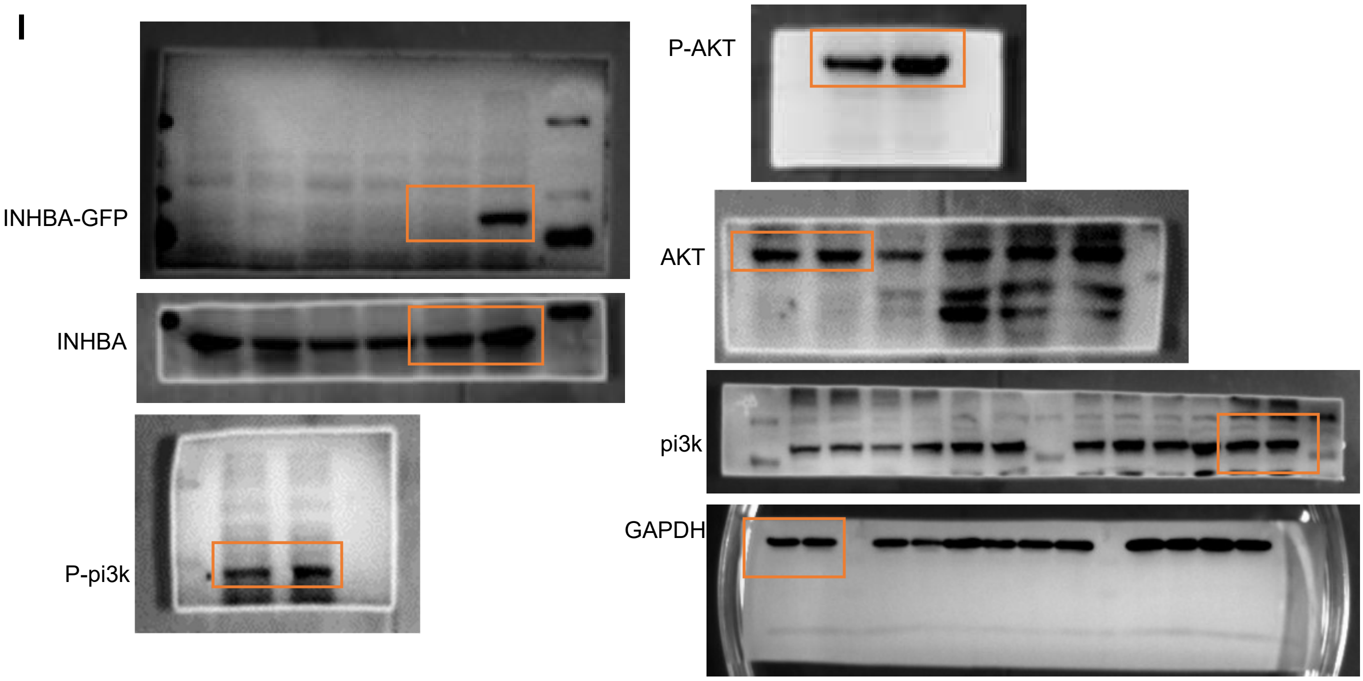
INHBA-GFP
INHBA
P-pi3k
P-AKT
AKT
pi3k
GAPDH

m

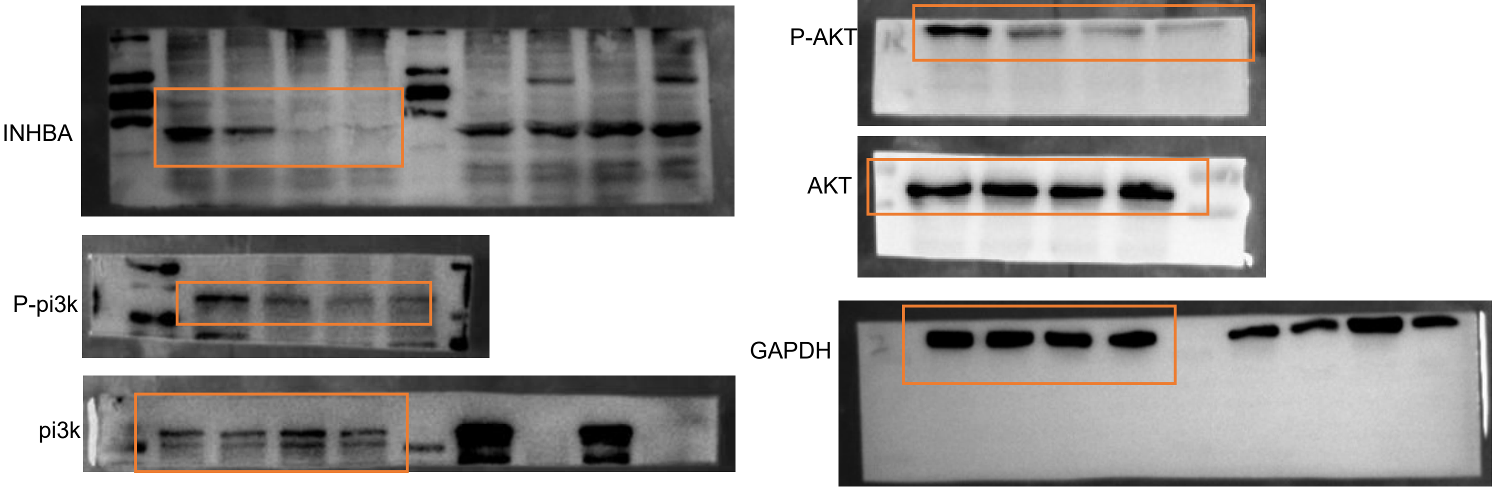
INHBA
P-pi3k
pi3k
P-AKT
AKT
GAPDH

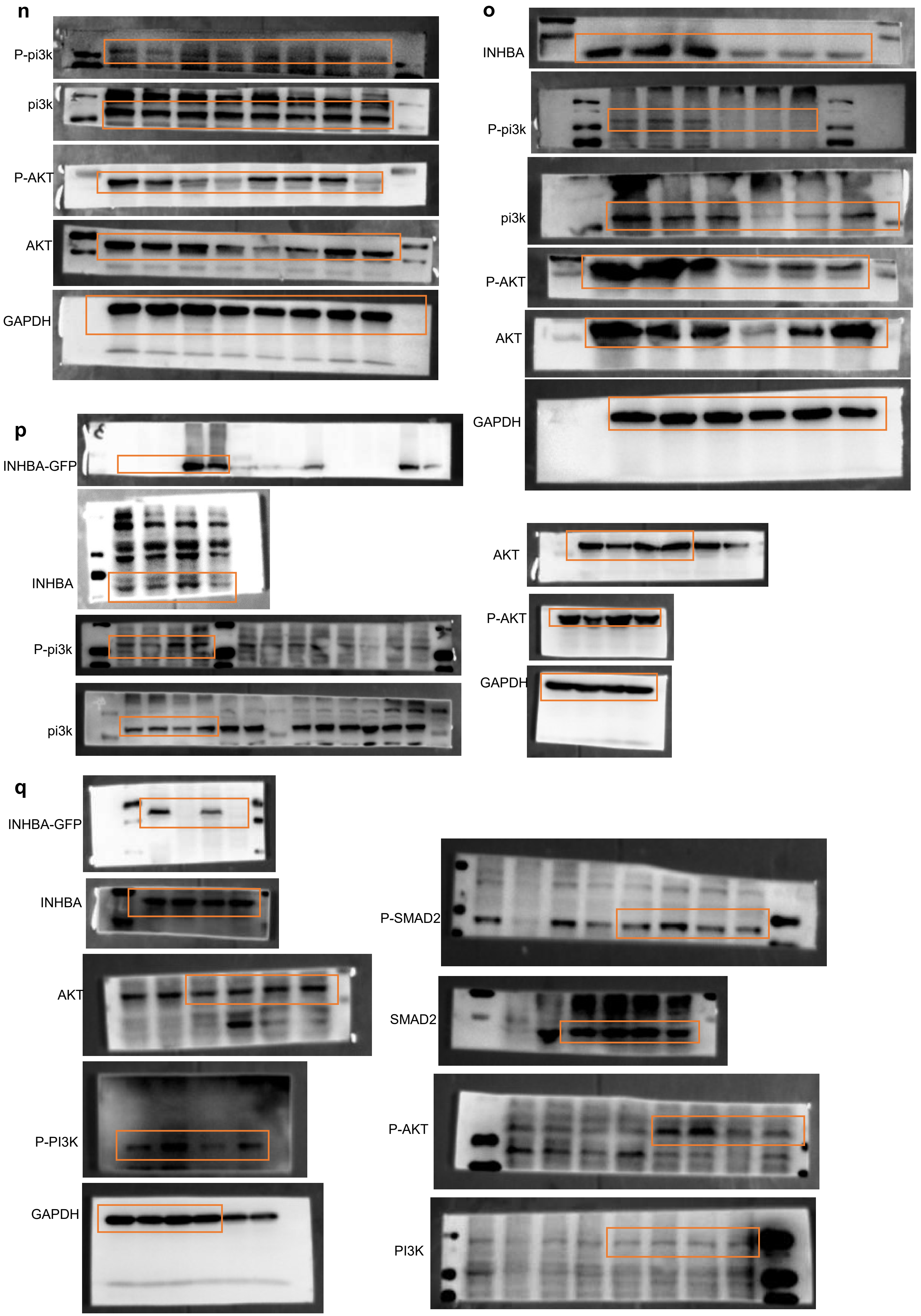
n
P-pi3k
pi3k
P-AKT
AKT
GAPDH
o
INHBA
P-pi3k
pi3k
P-AKT
AKT
GAPDH
AKT
P-AKT
GAPDH
p
INHBA-GFP
INHBA
P-pi3k
pi3k
q
INHBA-GFP
INHBA
AKT
P-PI3K
GAPDH
P-SMAD2
SMAD2
P-AKT
PI3K

r

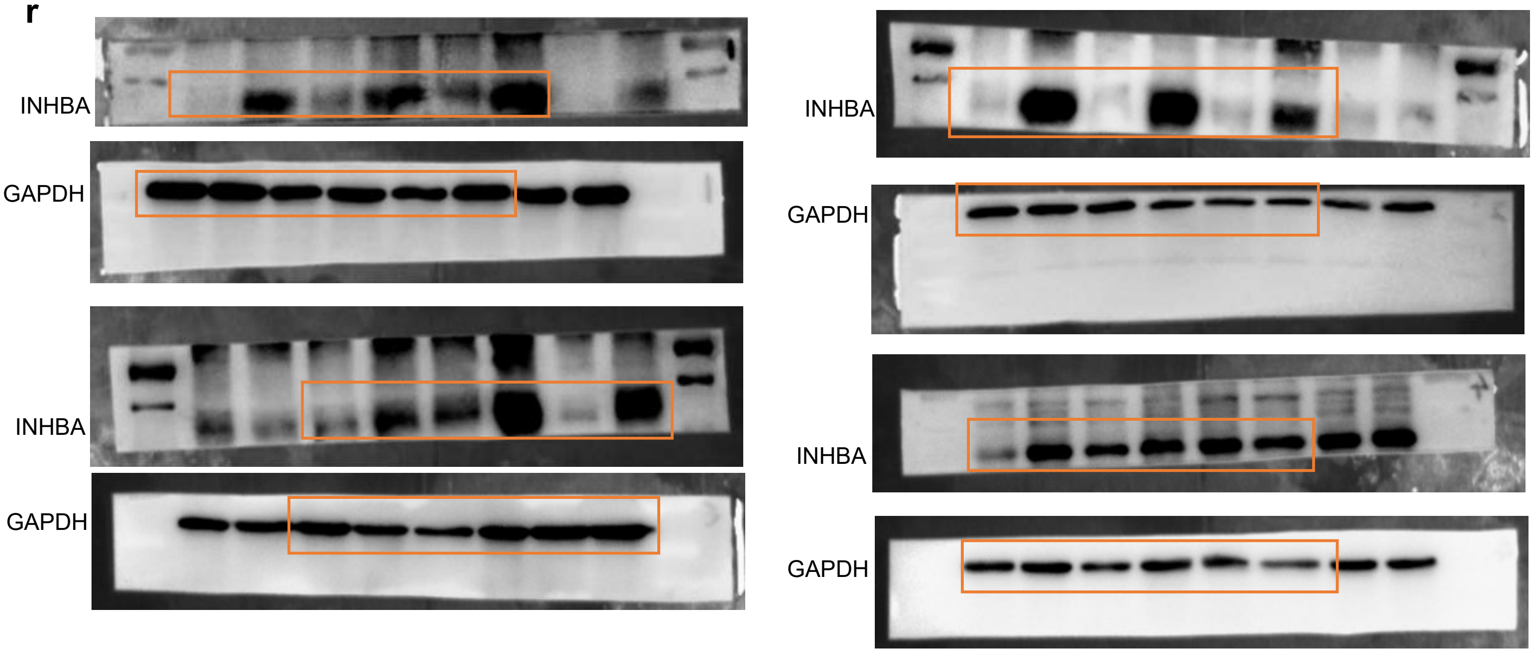

Figure S5 Original images of western blot (a-c) Original data of figure 3b,3d, and 3g 4f.**(d-e)** Original data of figure 4b, 4f. **(f-g)** Original data of figure 5a, 5g. **(h-k)** Original data of figure 6d-6g**.** **(l-q)** Original data of figure 7c-7h. (r) Original data of figure 8b.

Table S1 qPCR primers sequences

Gene	Primer sequence
**INHBA-QF**	GGCAAGTTGCTGGATTATAGTG
**INHBA-QR**	CTGAGAGTTGGGTACATCCTTT
**CyclinD1-QF**	GTCCTACTTCAAATGTGTGCAG
**CyclinD1-QR**	GGGATGGTCTCCTTCATCTTAG
**EGF-QF**	GAAGCATTGGACAAGTATGCAT
**EGF-QR**	CAGCTTCTGAGTCCTGTAGTAG
